# Dynamic Reconfiguration of Cognitive Networks and Recovery From Microlesion Effects in Parkinson's Disease: Insights From a Longitudinal fNIRS Study

**DOI:** 10.1002/cns.70835

**Published:** 2026-03-15

**Authors:** Xiang Wei, Yuting Tian, Qiutian Lu, Jingxuan Liu, Guanghan Lu, Jian Sun, Bei Luo, Liang Zhao, Chang Qiu, Wenwen Dong, Wenbin Zhang

**Affiliations:** ^1^ Department of Functional Neurosurgery The Affiliated Brain Hospital of Nanjing Medical University Nanjing City Jiangsu Province China

**Keywords:** deep brain stimulation, fNIRS, microlesion effect, Parkinson's disease, verbal fluency task

## Abstract

**Background:**

Bilateral subthalamic nucleus deep brain stimulation (STN‐DBS) significantly improves motor symptoms in advanced Parkinson's disease (PD). However, the perioperative “microlesion effect” (MLE) is often associated with cognitive dysfunction, notably declines in verbal fluency (VFT). The dynamic neural mechanisms underlying cognitive network impairment during the MLE phase and functional reorganization following DBS stimulation remain poorly understood.

**Aims and Methods:**

This study employed longitudinal task‐based functional near‐infrared spectroscopy (fNIRS) to prospectively track 20 PD patients undergoing bilateral STN‐DBS. To effectively disentangle the effects of natural surgical recovery from those specific to electrical stimulation, data were collected at four critical time points: 1 day preoperatively (Pre, T0), 7 days postoperatively (MLE phase, Post, T1), 1 month postoperatively with stimulation off (endpoint of natural recovery, Off, T2), and 1 week after stimulation onset (T3). VFT behavioral performance and global cognitive function (MoCA) were assessed concurrently. Hemodynamic signals from fNIRS were analyzed to examine activation changes in the prefrontal‐temporal cortices. Furthermore, graph theory analysis was applied to quantify the dynamic evolution of topological properties within the core cognitive and motor networks.

**Results:**

VFT scores dropped during MLE (8.70 ± 2.30 to 5.70 ± 1.78, *p* < 0.01), partially recovering post‐stimulation (8.15 ± 2.48, *p* < 0.05). MoCA scores also declined in MLE (25.40 ± 1.27 to 21.95 ± 1.10, *p* < 0.001). Neuroimaging showed activated channels decreased from 8 preoperatively to 2 during MLE (FDR‐corrected), followed by reactivation to 12 channels after stimulation, particularly in dorsolateral/ventrolateral prefrontal regions. Between‐group comparisons revealed enhanced activation in right DLPFC (Ch6), right SMA (Ch19), and left VLPFC (Ch47) after stimulation versus MLE (all *p* < 0.05, FDR‐corrected).

**Conclusion:**

Our findings indicate that MLE‐related cognitive decline may stem from acute local network disruption, while DBS can promote functional reorganization of cognitive networks. fNIRS proves to be a valuable tool for monitoring DBS‐induced neuroplasticity in PD.

## Graphical abstract

This longitudinal fNIRS study reveals that STN‐DBS induces a dynamic “suppression‐reorganization” of prefrontal‐temporal cognitive networks in Parkinson's patients. Cortical activation decreased during the microlesion period but rebounded beyond baseline after stimulation, with graph theory confirming reorganization independent of motor networks. fNIRS proves valuable for tracking DBS‐related neuroplasticity.

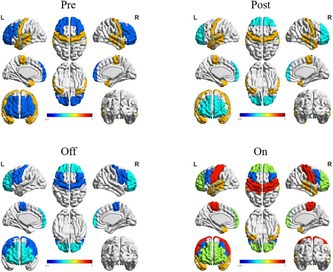

## 1 Introduction

Parkinson's disease (PD) is primarily characterized by motor symptoms such as bradykinesia, resting tremor, and rigidity [1]. Deep brain stimulation (DBS), a minimally invasive neuromodulation technique, can significantly ameliorate motor symptoms in movement disorders such as PD [2]. The improvement of such motor symptoms occurs not only after DBS activation but also manifests significantly approximately 1 week postoperatively, a phenomenon termed the microlesional effect (MLE) [3]. Notably, even without DBS stimulation being activated, the patients still demonstrated a significant improvement in motor scale scores at this time point [4]. However, alongside the significant improvement in motor symptoms during the postoperative microlesional effect period, the patients' cognitive scale scores conversely declined, with a prominent abnormality observed in the verbal fluency test (VFT) [5]. This phenomenon occurs after electrode implantation in the subthalamic nucleus (STN) and prior to the initiation of active stimulation. Although it is commonly observed in clinical practice, its underlying mechanism remains unclear. Accordingly, a growing number of researchers worldwide have begun to focus on changes in cognitive outcomes following DBS [6]. Numerous previous studies have demonstrated that patients with PD may experience deterioration in cognitive function and neuropsychiatric symptoms following DBS surgery [7, 8, 9], with a particularly prominent decline observed in VFT performance [10, 11]. A systematic review conducted by Mahesh wary [12] revealed that DBS is associated with declines in verbal fluency and attention; a randomized controlled trial (RCT) conducted by Leimbach [13] demonstrated a significant difference in VFT performance among patients with PD who underwent STN‐DBS, with a marked post‐operative decline in VFT scores. Previous studies have indicated that the decline in VFT performance may be directly associated with the surgery itself rather than the initiation of DBS stimulation [14]. A previous functional magnetic resonance imaging (fMRI) study by our group [15] demonstrated that the postoperative MLE in patients may be the primary cause of the decline in VFT performance. Specifically, the implantation of surgical electrodes significantly impacts three resting‐state functional networks associated with cognition—default mode network (DMN), executive control network (ECN), and dorsal attention network (DAN)—thereby leading to cognitive abnormalities.

In recent years, the rapid advancement of neuroimaging techniques such as fMRI, Diffusion Tensor Imaging (DTI), and Positron Emission Tomography‐Computed Tomography (PET‐CT) has provided novel methodologies and tools for investigating the neural mechanisms of PD. A longitudinal DTI‐based study in healthy human subjects revealed potential direct structural connectivity between the STN and the prefrontal cortex (PFC), particularly the dorsolateral prefrontal cortex (DLPFC) and ventrolateral prefrontal cortex (VLPFC). These connections form an integral component of the non‐motor fronto‐subthalamic‐thalamo‐cortical circuit [16]. A separate resting‐state functional magnetic resonance imaging (rs‐fMRI) study has also confirmed stable functional connectivity between the STN and cognitive‐related prefrontal cortical regions [17]. Collectively, these findings indicate that the STN acts as a nodal component of large‐scale brain networks under physiological conditions, and it not only mediates motor regulation but also is integrated into the cognitive network. This affords a solid theoretical foundation for investigating the modulatory effects and impacts of bilateral STN‐DBS on the cognitive network. Functional near‐infrared spectroscopy (fNIRS) power spectrum, an emerging non‐invasive, convenient, and cost‐effective brain functional imaging technique, differs from fMRI, which is constrained by strict acquisition conditions, susceptibility to head motion, and inability for ambulatory data collection. Specifically, fNIRS focuses on investigating the spatiotemporal changes in cerebral blood flow [18], based on which specific hemodynamic curves are generated to characterize the activation effect and magnitude of relevant brain regions. Moreover, fNIRS has gained widespread recognition among scholars worldwide owing to its unique advantages, including simplicity, portability, strong anti‐interference capability, and high temporal resolution [19, 20]. Currently, it has been widely applied in various neurological disorders such as PD and Alzheimer's disease (AD), particularly in the assessment of cognitive function [21, 22].

However, no relevant studies have yet elucidated the cerebral hemodynamic changes and activation patterns in patients before and after STN‐DBS as well as following the initiation of stimulation, nor have they explored the potential mechanisms underlying the decline in cognitive executive function during the postoperative MLE period. Against this background, we utilized fNIRS to conduct studies on prefrontal‐temporal brain networks in patients receiving bilateral STN‐DBS, aiming to explore changes in brain region activation levels in these patients during the perioperative period of bilateral STN‐DBS. Task‐state fNIRS‐VFT experiments were performed separately at four time points: 1 day before surgery (Pre, T0), 7 days after surgery (Post, MLE, T1), 1 month after surgery with stimulation turned off (Off, T2), and 1 week after stimulation was turned on at 1 month postoperatively (On, T3). Specifically, we evaluated differences in brain activation levels among PD patients at these four time points, simultaneously investigated changes in cognitive networks and related network topological indicators of PD patients during the perioperative period, verified changes in cerebral blood perfusion during the MLE period on this basis, and explored potential mechanisms underlying the impairment of cognitive executive function in these patients.

## 2 Materials and Methods

### 2.1 Participants

Twenty‐three patients diagnosed with idiopathic PD were initially enrolled in this study. The diagnosis of PD was based on the UK Parkinson's Disease Society Brain Bank clinical diagnostic criteria [23] and the Movement Disorder Society (MDS) PD clinical diagnostic criteria [23, 24]. All patients met the surgical indications [25] and underwent DBS surgery. Patients with other severe central nervous system diseases, those taking drugs that affect brain function (e.g., antipsychotics), and those with contraindications to magnetic resonance imaging (MRI) were excluded. This study was approved by the hospital ethics committee, and all patients signed a written informed consent form before participating in the experiment. Finally, 20 patients were included in the study, while 3 patients were excluded due to failure to complete the 1‐month postoperative follow‐up.

### 2.2 Clinical Assessment

In this study, all PD patients underwent scale assessments (all in the off‐state) and task‐state fNIRS‐VFT tests at four time points: 1 day before surgery (Pre, T0), 7 days after surgery (Post, T1), 1 month after surgery with stimulation not turned on (Off, T2, the endpoint of natural recovery), and 1 week after stimulation was turned on (On, T3, after stimulation intervention). The off‐state was defined as withdrawal of dopaminergic drugs for at least 12 h to reduce the impact of drugs on data collection. This staging design could effectively separate postoperative natural recovery from stimulation‐specific effects: T2, as the endpoint of natural recovery, could exclude acute surgical effects such as microdamage and edema; the comparison between T3 and T2 could be directly attributed to electrical stimulation intervention; the comparison between T0 and T2 could evaluate the baseline changes of cognitive networks after natural recovery. In addition, the Unified Parkinson's Disease Rating Scale III (UPDRS III) Item [26] was used to assess motor symptoms. Furthermore, the Hamilton Anxiety Scale (HAMA) and Hamilton Depression Scale (HAMD) were used to evaluate the severity of anxiety and depression, respectively, and the Montreal Cognitive Assessment (MoCA) was used to assess cognitive status. Task‐state fNIRS‐VFT data were collected at T0, T1, T2, and T3, respectively, and the number of words generated during the VFT task was recorded simultaneously.

### 2.3 Surgery

All patients underwent bilateral STN‐DBS under general anesthesia. Preoperative navigational planning was performed using the Leksell Stereotactic System. Bilateral electrode implantation (Model L301, PINS Medical Co. Ltd., Beijing, China) was conducted in accordance with the preoperative plan. Intraoperative electrophysiological monitoring was carried out using the Omega recording system to identify the characteristic electrophysiological patterns of the STN, ensuring the accuracy of electrode placement. Postoperative computed tomography (CT) scan was performed on the first postoperative day, which was fused with preoperative MRI to confirm the precise positioning of the electrodes. All patients achieved satisfactory surgical incision healing postoperatively.

### 2.4 FNIRS Acquisition

**FIGURE 1:**
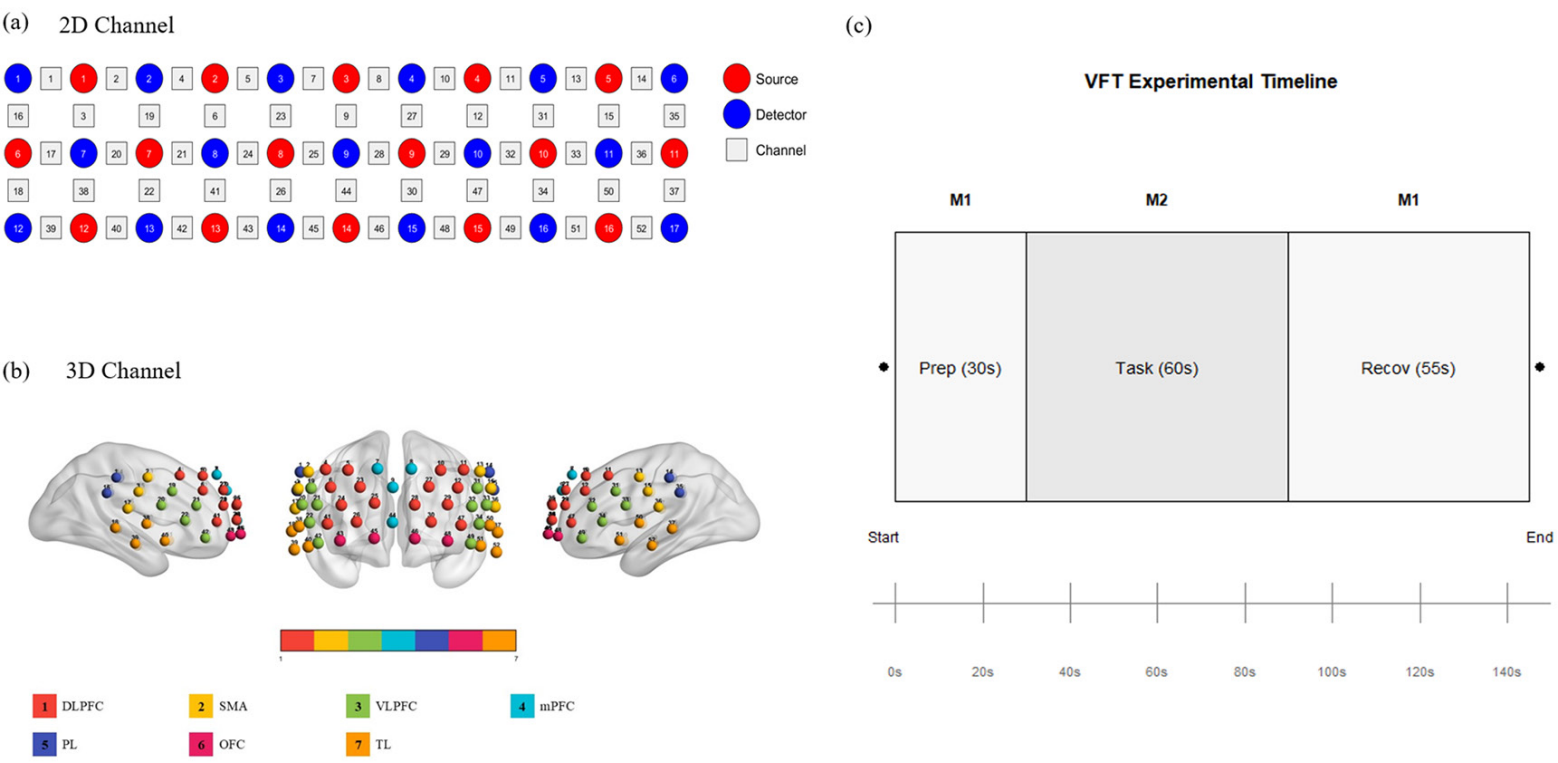
Diagrams of Channel Arrangement and Experimental Paradigm of the VFT. (a) 2D diagram of channel arrangement. 3 × 11 cross‐orthogonal configuration consisting of 16 emitters and 17 detectors, forming 52 measurement channels. Red circles represent light sources, blue circles represent detectors, and white squares represent channels. (b) 3D diagram of channel arrangement. A total of 52 channels corresponding to different brain regions including the prefrontal cortex and bilateral temporal lobes. (c) Experimental Paradigm of the VFT. M1 (Marker 1)‐Preparation phase‐Participants counted numbers softly to maintain a stable state (duration: 30 s); M2 (Marker 2)‐Task phase‐A Chinese character was presented every 20 s, and participants were required to generate as many words as possible using the presented character (total duration: 60 s). The number of correctly generated words was recorded; M1 (Marker 1)‐Recovery phase—Participants counted numbers softly to restore a stable state (duration: 55 s).

In the present study, fNIRS optical imaging system (BrainScan‐N3001F, MindArk BioMedical Technology Co. Ltd., Beijing, China) consisting of 16 emitter probes and 17 detector probes was utilized. These probes were alternately arranged to form 52 measurement channels, covering the prefrontal cortex and bilateral temporal lobes. The distance between adjacent light sources and probes was 3 cm, with a sampling frequency of 18 Hz. The modified Beer–Lambert law was employed to monitor changes in the concentrations of oxyhemoglobin (HbO_2_) and deoxyhemoglobin (HbR) in the cerebral cortex. The fNIRS cap was designed based on the international 10–20 electrode placement system. Based on the modified Beer–Lambert law [27], this system measures the relative changes in HbO_2_ and HbR using three wavelengths: 780, 808, and 830 nm. In accordance with the international 10–20 system for electroencephalography (EEG), the upper edge of the lowest probe was placed along the T4—Fpz—T3 line. To obtain the corresponding relationship between measurement channels and cerebral cortical positions, a virtual registration method based on the international 10–20 system was adopted [28, 29]: (1)We mapped the channels of our fNIRS system to those reported by Hitachi, thereby obtaining the specific coordinates of each channel; (2) Referring to the standard fNIRS channel template provided by Takizawa et al. [30], the preset 3D coordinates of 52 channels in the MNI space were obtained; (3) The MNI coordinates of 52 channels were one‐to‐one registered to a standard 3D brain template using the virtual registration algorithm implemented in the NIRS‐KIT software package (Version 3.0), and the nearest neighbor method was adopted to determine the brain region corresponding to each channel. Its accuracy and reliability have been verified in previous studies [31]. According to the above mapping results, the cerebral regions covered by the 52 fNIRS channels included: bilateral dorsolateral prefrontal cortex (DLPFC) [Ch 4, 5, 6, 10, 11, 12, 23, 24, 25, 26, 27, 28, 29, 30, 41 and 47], ventrolateral prefrontal cortex (VLPFC) [Ch 19, 20, 21, 22, 31, 32, 33, 34, 42 and 49], medial prefrontal cortex (mPFC) [Ch 7, 8, 9, 44], orbitofrontal cortex (OFC) [Ch 43, 45, 46, 48], temporal lobe (TL) [Ch 18, 37, 38, 39, 40, 50, 51, 52], sensorimotor areas [Ch 2, 3, 13, 15, 17, 36], and inferior parietal lobule (IPL) [Ch 1, 14, 16, 35], which is consistent with previous fNIRS studies [31, 32]. Since the lateral orbitofrontal cortex is generally considered a component of the VLPFC, Ch 42 and 49 were classified as part of the VLPFC [33] (Figure 1).

### 2.5 Task Description

fNIRS device was placed in a quiet, dimly lit room and warmed up prior to testing to minimize systematic signal drift and ensure more stable signal acquisition [34]. The device was positioned on the side of the participants' heads. Before the initiation of testing, participants were seated in a chair with a reclined backrest for comfort and instructed to fix their gaze on a “+” symbol displayed on the wall directly in front of them. The VFT performance was utilized to assess executive function. Prior to the task, participants were guided to rest quietly and comfortably for 5 min to acclimatize to the environment and alleviate anxiety. During the task, participants were instructed to maintain focus on the “+” symbol to reduce head or eye movements and associated artifacts.

The task‐based fNIRS‐VFT protocol consisted of the following phases: a 30‐s baseline period, during which participants were required to remain still and silent while repeatedly counting numbers 1–5; subsequent task periods, wherein participants were presented with three Chinese characters (“Hong” [red], “Da” [large], and “Hai” [sea]) at 20‐s intervals [35]. during each 20‐s interval, participants were asked to generate as many words as possible related to the presented character until the next character was announced; and a final 55‐s baseline period, again involving repeated counting of numbers 1–5. VFT performance data were collected by recording the total number of correctly generated words per participant (Figure 1).

### 2.6 Data Preprocessing

In the present study, a prospective self‐controlled design was adopted. Clinical scale data were integrated to analyze 20 patients at three time points: 1 day before surgery (Pre, T0), 7 days after surgery (Post, T1), 1 month after surgery with stimulation not turned on (Off, T2, the endpoint of natural recovery), and 1 week after stimulation was turned on (On, T3, after stimulation intervention). The fNIRS data were preprocessed using the NIRS‐KIT (Version 3.0) software package [36]. The primary objective of preprocessing was to remove noise from the raw data and retain, as much as possible, the signal components corresponding to blood oxygenation changes induced by neural activity [37]. First, the raw intensity data were converted into optical density (OD) data with a differential pathlength factor (DPF) of 6. Subsequently, the data were truncated to retain the time window of −10 to 115 s relative to Marker 2, which encompasses the entire task phase and recovery phase. Finally, first‐order detrending and temporal derivative distribution repair (TDDR) were performed to eliminate potential artifacts introduced during data acquisition [38]. Subsequently, a band‐pass filter with a frequency range of 0–0.1 Hz was applied to eliminate the effects of physiological and systematic noise. Finally, the optical density values were converted into the relative concentration changes of mean HbO_2_ and HbR. Given that the HbO_2_ signal is more sensitive to regional cerebral blood flow (rCBF) than the HbR signal, the mean HbO_2_ was selected as the hemodynamic response index [39].

The mean HbO_2_ of each patient was analyzed using a general linear model (GLM), with a canonical hemodynamic response function (HRF) convolved with the task duration. The baseline was defined as the 30‐s period prior to task initiation and the 55‐s period post‐task. GLM parameters in each channel were estimated to derive activation beta values (*β*‐values) under each condition. Task‐related *β*‐values were calculated as the *β*‐values during the word‐generation process minus the baseline *β*‐values. Finally, the results were visualized using BrainNet Viewer [40].

Statistical analyses were performed using SPSS 27.0.1, and data visualization was conducted with GraphPad Prism 10. To assess statistical differences in demographic characteristics, clinical variables, and behavioral data during the VFT task among the four groups, appropriate analytical methods were employed, with a two‐tailed *p* < 0.05 considered statistically significant. The selection of analytical methods was initially based on data type classification, normality tests, and homogeneity of variance tests. All inter‐group comparisons were analyzed first using one‐way analysis of variance (one‐way ANOVA), followed by paired *t*‐test; these analyses and correlation analyses were corrected for False Discovery Rate (FDR), with a corrected *p* < 0.05 considered statistically significant. All *p*‐values reported in this study are the corrected results. Pearson correlation analysis, partial correlation analysis, and graph theory analysis of regions of interest were performed using R software to explore the correlations between the average ΔHbO2, VFT behavioral counts, and MoCA scores.

## 3 Results

### 3.1 Demographic and Clinical Scale Analyses

Finally, a total of 20 patients with idiopathic PD who underwent bilateral STN‐DBS were enrolled in this study. The demographic and clinical characteristics of the enrolled patients are summarized in Table 1. No significant differences were observed among the four groups in age, gender, years of education, disease severity (Marschall‐Hoehn & Yahr [M‐H&Y] stage), as well as scores on the HAMA and HAMD (all *p* > 0.05). However, patients in the T1 (post‐DBS) group exhibited a significant decrease in both MoCA and VFT scores compared with those in the pre‐DBS T0 group (both *p* < 0.001). In the T2 (stimulation‐off) group, MoCA and VFT scores were significantly recovered in the stimulation‐naive state, yet still remained significantly lower than the pre‐operative T0 baseline (*p* < 0.01). Following electrical stimulation administration in the T3 (stimulation‐on) group, patients' MoCA and VFT scores continued to be significantly lower than the pre‐operative baseline (both *p* < 0.05), with no statistically significant difference detected relative to the T2 stage (*p* > 0.05). All the above results are presented in Table 1.

**TABLE 1:** Demographic data.

	*N* = 20 (Mean ± SD)	*p*
Gender(Men/Women)	6/14	/
Age(years)	70.95 ± 6.62	/
Years of education (years)	9.80 ± 2.33	/
M‐H&Y	3.0 (2.63, 4.00)	/
HAMA	6.70 ± 1.38	/
HAMD	6.15 ± 1.18	/
MoCA		< 0.0001^a^
T0(Pre)	25.40 ± 1.27	< 0.01^b^
T1(Post)	21.95 ± 1.10	< 0.0001^c^
T2(Off)	24.05 ± 1.32	ns^d^
T3(On)	24.20 ± 1.06	< 0.0001^e^
		< 0.0001^f^
VFT performance		< 0.05^a^
		< 0.0001^b^
T0(Pre)	8.70 ± 2.30	< 0.0001^c^
T1(Post)	5.70 ± 1.78	ns^d^
T2(Off)	7.25 ± 1.89	< 0.001^e^
T3(On)	8.15 ± 2.48	< 0.001^f^

*Note:* Data meeting normal distribution were expressed as mean ± standard deviation (x ± s), while data that did not meet normal distribution were presented as median and interquartile range.

Abbreviation: M‐H&Y, Modified Hoehn and Yahr Scale.

^a^ pre vs on.

^b^ pre vs off.

^c^ pre vs post.

^d^ on vs off.

^e^ on vs post.

^f^ post vs off.

### 3.2 Brain Activation Patterns During VFT Across Different Groups

**FIGURE 2:**
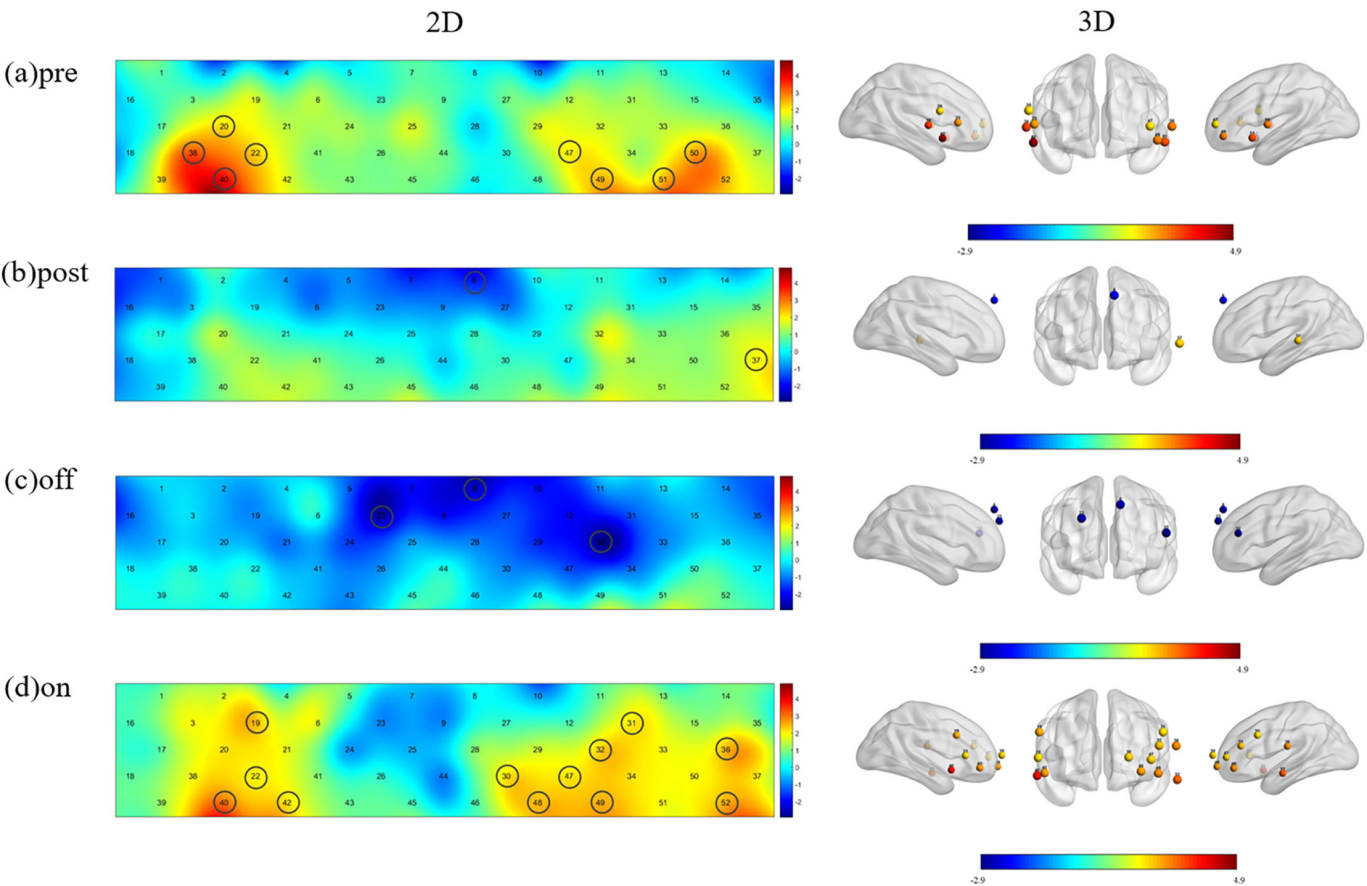
Brain activation patterns during the VFT across different groups. (a) Preoperative group (T0, Pre): Left panel = 2D map of oxyhemoglobin (HbO_2_) activation; Right panel = 3D map of HbO_2_ activation. Significantly activated channels (*p* < 0.05, false discovery rate [FDR]‐corrected) included Ch20, Ch22, Ch38, Ch40, Ch47, Ch49, Ch50, and Ch51. (b) Postoperative microlesion period group (T1, Post): Left panel = 2D map of HbO_2_ activation; Right panel = 3D map of HbO_2_ activation. Significantly activated channels (*p* < 0.05, FDR‐corrected) included Ch8 and Ch37. (c) End of the natural recovery phase at 1 month postoperatively without electrical stimulation initiation (T2, Off): Left panel = 2D map of HbO_2_ activation; Right panel = 3D map of HbO_2_ activation. Significantly activated channels (*p* < 0.05, FDR‐corrected) included Ch, Ch23 and Ch32. (d) DBS‐activated group (T3, On): Left panel = 2D map of HbO_2_ activation; Right panel = 3D map of HbO_2_ activation. Significantly activated channels (*p* < 0.05, FDR‐corrected) included Ch19, Ch22, Ch30, Ch31, Ch32, Ch36, Ch40, Ch42, Ch47, Ch48, Ch49, and Ch52. Activation intensity is represented by a color gradient from blue (low activation) to red (high activation).

GLM analyses were performed on participant data from the T0 (pre), T1 (post), T2 (off), and T3 (on) groups separately to calculate the beta (*β*) value for each channel, and task‐related activations of all channels across the four groups were identified. Data from 20 participants were included in the analysis for each group, with the results as follows: (1) T0: Significant HbO2 activations were observed in 8 channels (Ch20, Ch22, Ch38, Ch40, Ch47, Ch49, Ch50, Ch51; *p* < 0.05, FDR‐corrected). These channels were primarily localized to the prefrontal and temporal lobes, including the bilateral DLPFC (left: Ch47, Ch49; right: Ch20, Ch22) and bilateral TL (left: Ch50, Ch51; right: Ch38, Ch40). (2) T1: Only 2 channels (Ch8, Ch37; *p* < 0.05, FDR‐corrected) exhibited task‐related significant activations, corresponding to the left superior frontal gyrus and left middle temporal gyrus, as well as the functional brain regions of the mPFC (Ch8) and left TL (Ch37), respectively. (3) T2: Significant HbO_2_ activations were detected in 3 channels (Ch8, Ch23, Ch32; *p* < 0.05, FDR‐corrected), encompassing the mPFC (Ch8), right DLPFC (Ch23), and left ventrolateral prefrontal cortex (VLPFC) (Ch32). (4) T3: Extensive activations were observed, with 12 channels showing significant task‐related HbO2 activations (Ch19, Ch22, Ch30, Ch31, Ch32, Ch36, Ch40, Ch42, Ch47, Ch49, Ch52; *p* < 0.05, FDR‐corrected). These channels covered the prefrontal lobe, temporal lobe, and sensorimotor areas, corresponding to the bilateral SMA (left: Ch36; right: Ch19), bilateral VLPFC (left: Ch47, Ch49; right: Ch22, Ch42), left DLPFC (Ch30, Ch31, Ch48), and bilateral TL (left: Ch52; right: Ch40). All results are illustrated in Figure 2 below.

### 3.3 Differences in Brain Activation During the VFT Across Groups

**FIGURE 3:**
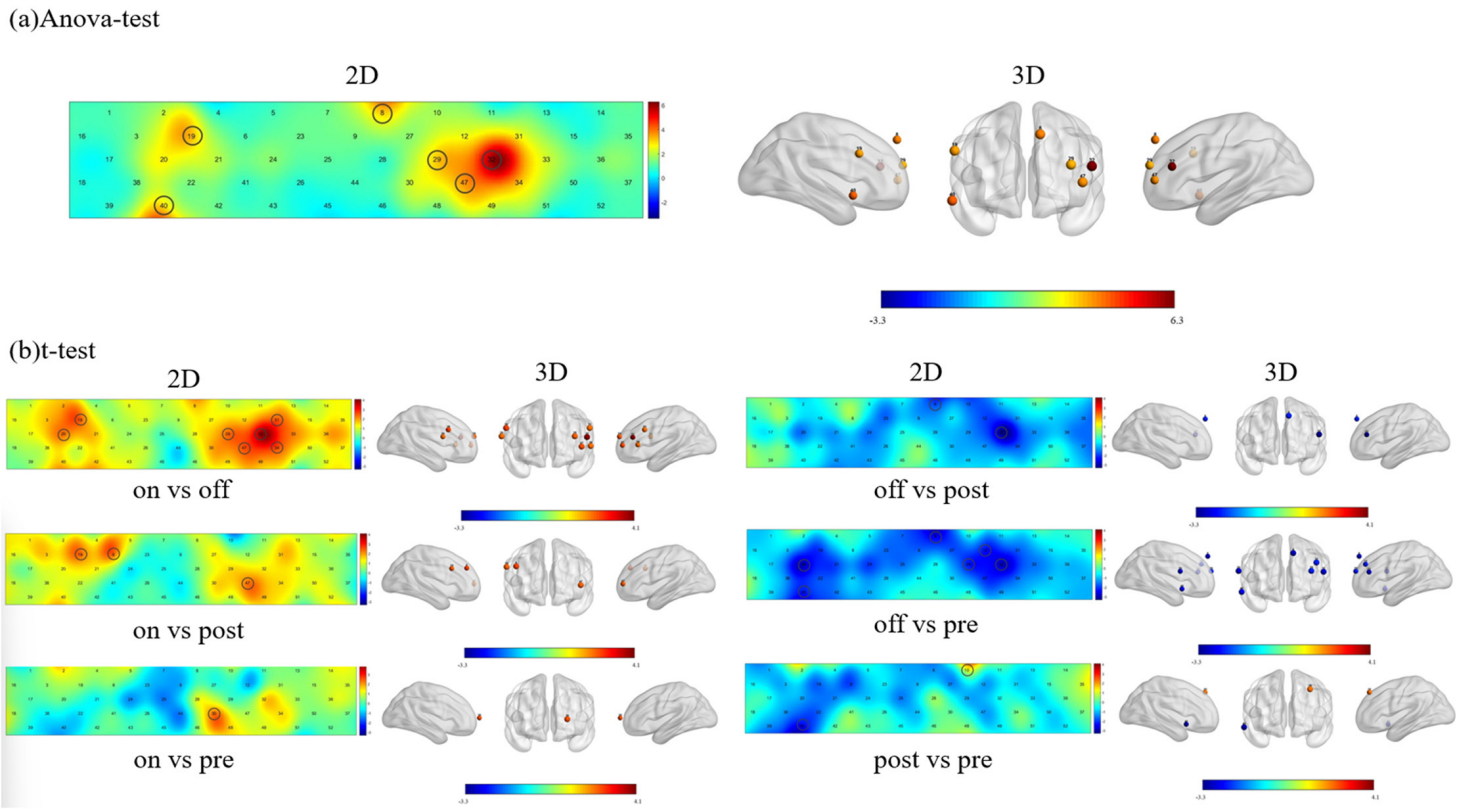
Differences in brain activation during the VFT across different groups. (a) Repeated‐measures analysis of variance (ANOVA) was conducted on the data from the four participant groups, and a significant main effect was identified for Ch8, Ch19, Ch29, Ch32, Ch40 and Ch47 across the four groups (*p* < 0.05, FDR‐corrected). These channels corresponded to the medial prefrontal cortex (mPFC, Ch8), ventrolateral prefrontal cortex (VLPFC; right: Ch19, left: Ch32), left dorsolateral prefrontal cortex (DLPFC, Ch29, Ch47), and right temporal lobe (TL, Ch40). These findings confirmed that significant activation differences were present in the mPFC, VLPFC, left DLPFC and right TL across the four distinct time points, and these brain regions were highly task‐relevant. Left panel = 2D map of oxyhemoglobin (HbO_2_) activation comparison; Right panel = 3D map of activation comparison. (b) Paired‐samples *t*‐test were conducted on the data from the four participant groups to assess intergroup differences, with detailed results reported in the main text. Left panel = 2D map of HbO_2_ activation comparison; Right panel = 3D map of activation comparison.

Repeated‐measures analysis of variance (ANOVA) was first performed on participant data from the four groups to examine intergroup differences in task‐related channel activations during the VFT task. Significant main effects were observed for Ch8, Ch19, Ch29, Ch32, Ch40, and Ch47 across the four groups (all *p* < 0.05, FDR‐corrected), which corresponded to the mPFC (Ch8), VLPFC (right: Ch19; left: Ch32), left DLPFC (Ch29, Ch47), and right TL (Ch40). This finding indicated that significant differences in task‐related activations existed in the mPFC, VLPFC, left DLPFC, and right TL across the four time points, with these cortical regions being highly correlated with the VFT task performance. Subsequent simple effect analysis with pairwise paired‐samples *t*‐test was conducted to further explore intergroup differences in task‐related channel activations, yielding the following results: (1) T1 versus T0 (Post vs. Pre): Significant differences were detected in Ch10 (left DLPFC) and Ch40 (right TL) (*p* < 0.05, FDR‐corrected). Specifically, the left DLPFC exhibited significantly enhanced activation in the Post group relative to the Pre group, whereas activation of the right TL was significantly attenuated; (2) T2 versus T1 (Off vs. Post): Significant differences were observed in Ch8 (mPFC) and Ch32 (left VLPFC) (*p* < 0.05, FDR‐corrected), with the Off group showing significantly weaker activations in the mPFC and left VLPFC compared to the Post group; (3) T3 versus T1 (On vs. Post): Significant differences were found in Ch6 (right DLPFC), Ch19 (right SMA), and Ch47 (left VLPFC) (*p* < 0.05, FDR‐corrected). The On group exhibited significantly higher activation levels in the right DLPFC, right SMA, and left VLPFC relative to the Post group; (4) T3 versus T0 (On vs. Pre): A significant difference was detected in Ch30 (left DLPFC) (*p* < 0.05, FDR‐corrected), with the On group showing significantly enhanced activation in this channel compared to the Pre group; (5) T2 versus T0 (Off vs. Pre): Significant differences were identified in Ch8 (mPFC), Ch12/29 (left DLPFC), Ch20 (right VLPFC), Ch32 (left VLPFC), and Ch40 (right TL) (*p* < 0.05, FDR‐corrected). Activations in the mPFC, bilateral VLPFC, left DLPFC, and right TL were significantly weaker in the Off group relative to the Pre group; (6) T3 versus T2 (On vs. Off): Significant differences were observed in Ch19/20 (right VLPFC), Ch29/47 (left DLPFC), and Ch31/32/34 (left VLPFC) (*p* < 0.05, FDR‐corrected). The On group displayed significantly higher activation levels in the bilateral VLPFC and left DLPFC compared to the Off group. All intergroup difference results are illustrated in Figure 3.

### 3.4 Correlation Analysis Between Brain Activation Intensity and Clinical Scale Scores During the VFT Task

**FIGURE 4:**
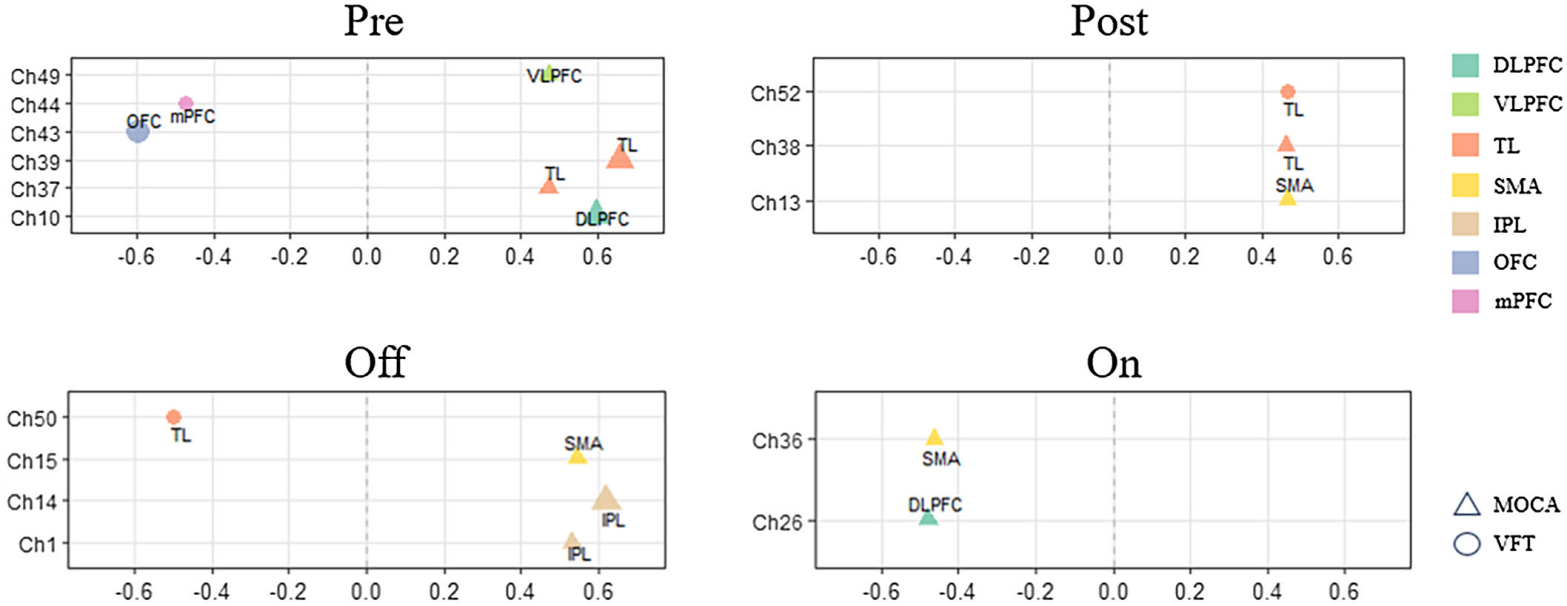
Partial Correlation Analyses Between HbO_2_ *β*‐Values, VFT Scores, and MoCA Scores (Adjusted for Years of Education): Partial correlation analyses, adjusting for years of education, were conducted across 52 fNIRS channels at four time points: (1) T0 (Pre): Positive correlations were observed between VFT scores and HbO_2_ *β* values in Ch10 (left DLPFC, *p* = 0.007, *r* = 0.596), Ch37 (left TL, *p* = 0.041, *r* = 0.473), Ch39 (right TL, *p* = 0.002, *r* = 0.659), and Ch49 (left VLPFC, *p* = 0.041, *r* = 0.473). Negative correlations were identified between MoCA scores and HbO_2_ *β* values in Ch43 (right DLPFC, *p* = 0.007, *r* = −0.597) and Ch44 (mPFC, *p* = 0.041, *r* = −0.472); (2) T1 (Post): Positive correlations were detected between VFT scores and HbO_2_ *β* values in Ch13 (left SMA, *p* = 0.045, *r* = 0.467) and Ch38 (right TL, *p* = 0.047, *r* = 0.462). A positive correlation was found between MoCA scores and HbO_2_ *β* values in Ch52 (left TL, *p* = 0.044, *r* = 0.466); (3) T2 (Off): Positive correlations were observed between VFT scores and HbO_2_ *β* values in Ch1 (right IPL, *p* = 0.019, *r* = 0.531), Ch14 (left IPL, *p* = 0.005, *r* = 0.621), and Ch15 (left SMA, *p* = 0.016, *r* = 0.545). A negative correlation was identified between MoCA scores and HbO_2_ *β* values in Ch50 (left TL, *p* = 0.023, *r* = −0.498); (4) T3 (On): Negative correlations were detected between VFT scores and HbO_2_ *β* values in Ch26 (right DLPFC, *p* = 0.038, *r* = −0.480) and Ch36 (left SMA, *p* = 0.046, *r* = −0.463). Circles represent VFT scores and triangles represent MoCA scores, with the larger area indicating stronger statistical significance and correlation strength.

Partial correlation analyses were conducted between the *β* values of HbO_2_ extracted from all channels via the GLM and behavioral scale scores. After controlling for years of education and undergoing FDR multiple correction, these analyses revealed significant associations between the HbO_2_ *β* values of specific channels and scores on cognitive‐related scales including the VFT and MoCA. The detailed results are as follows: (1) T0 (Pre): Positive correlations were observed between VFT scores and HbO_2_ *β* values in Ch10 (left DLPFC, adjust‐*p* = 0.007, *r* = 0.596), Ch37 (left TL, adjust‐*p* = 0.041, *r* = 0.473), Ch39 (right TL, adjust‐*p* = 0.002, *r* = 0.659), and Ch49 (left VLPFC, adjust‐*p* = 0.041, *r* = 0.473). Negative correlations were identified between MoCA scores and HbO_2_ *β* values in Ch43 (right DLPFC, adjust‐*p* = 0.007, *r* = −0.597) and Ch44 (mPFC, adjust‐*p* = 0.041, *r* = −0.472); (2) T1 (Post): Positive correlations were detected between VFT scores and HbO_2_ *β* values in Ch13 (left SMA, adjust‐*p* = 0.045, *r* = 0.467) and Ch38 (right TL, adjust‐*p* = 0.047, *r* = 0.462). A positive correlation was found between MoCA scores and HbO_2_ *β* values in Ch52 (left TL, adjust‐*p* = 0.044, *r* = 0.466); (3) T2 (Off): Positive correlations were observed between VFT scores and HbO_2_ *β* values in Ch1 (right IPL, adjust‐*p* = 0.019, *r* = 0.531), Ch14 (left IPL, adjust‐*p* = 0.005, *r* = 0.621), and Ch15 (left SMA, adjust‐*p* = 0.016, *r* = 0.545). A negative correlation was identified between MoCA scores and HbO_2_ *β* values in Ch50 (left TL, adjust‐*p* = 0.030, *r* = −0.498); (4) T3 (On): Negative correlations were detected between VFT scores and HbO_2_ *β* values in Ch26 (right DLPFC, adjust‐*p* = 0.038, *r* = −0.480) and Ch36 (left SMA, adjust‐*p* = 0.046, *r* = −0.463). All aforementioned results are presented in Table 2 and Figure 4 below.

**TABLE 2:** Partial correlation analyses between HbO_2_ *β*‐Values, VFT scores, and MoCA scores (adjusted for years of education).

	Group	Channel	*r*	Adjust‐*p*	Brain area
VFT	Pre	Ch10	0.596	0.007	left DLPFC
		Ch37	0.473	0.041	left TL
		Ch39	0.659	0.002	right TL
		Ch49	0.473	0.041	left VLPFC
	Post	Ch13	0.467	0.045	left SMA
		Ch38	0.462	0.047	right TL
	Off	Ch1	0.531	0.019	right IPL
		Ch14	0.621	0.005	left IPL
		Ch15	0.545	0.016	left SMA
	On	Ch26	−0.48	0.038	right DLPFC
		Ch36	−0.463	0.046	left SMA
MOCA	Pre	Ch43	−0.597	0.007	right DLPFC
		Ch44	−0.472	0.041	mPFC
	Post	Ch52	0.466	0.044	left TL
	Off	Ch50	−0.498	0.03	left TL
	On	/	/	/	/

### 3.5 Changes in Topological Metrics, and Intra‐Network Activation, Reorganization and Segregation of the Cognitive and Motor Core Networks During the VFT Task: A Graph Theory‐Based Study

**FIGURE 5:**
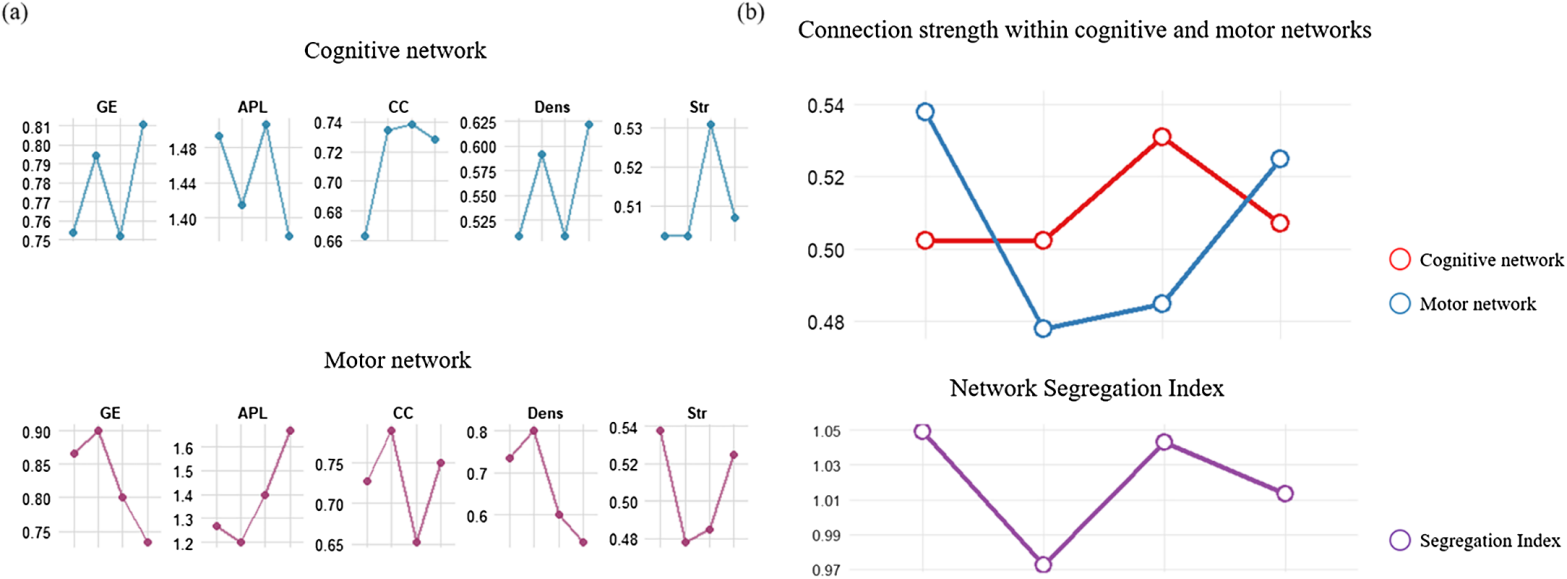
Changes in topological metrics and intra‐network connectivity strength of cognitive and motor networks during the perioperative period of DBS (T0: Pre, T1: Post, T2: Off, T3: On). (a) Topological metrics of core networks (global efficiency [GE], average shortest path length [APL], clustering coefficient [CC], density [Dens], strength [Str]): For the core cognitive network (including the DAN, VAN, TL, and IPL), GE was 0.754 pre‐operatively, slightly increased to 0.795 post‐operatively, recovered to the pre‐operative level (0.752) in the stimulation‐off phase, and then rose significantly to a stable level of 0.811 after stimulation initiation; APL slightly decreased from 1.494 pre‐operatively to 1.415 post‐operatively, rebounded to 1.506 in the stimulation‐off phase, and declined slightly to 1.379 after stimulation initiation; CC increased significantly from 0.663 pre‐operatively to 0.735 post‐operatively, rose slightly to 0.738 in the stimulation‐off phase, and decreased marginally to approximately 0.728 after stimulation initiation; Dens increased markedly from 0.509 pre‐operatively to 0.591 post‐operatively, fell back to 0.508 in the stimulation‐off phase, and rose to 0.621 after stimulation initiation; Str slightly increased from 0.502 pre‐operatively to 0.503 post‐operatively, rose to 0.531 in the stimulation‐off phase, and recovered to 0.507 after stimulation initiation. Overall, the cognitive network exhibited mild and transient fluctuations post‐operatively. For the motor network: GE increased from 0.867 pre‐operatively to 0.90 post‐operatively, decreased to 0.80 in the stimulation‐off phase, and continued to drop to 0.733 after stimulation initiation; APL slightly decreased from 1.267 pre‐operatively to 1.20 post‐operatively, recovered to 1.40 in the stimulation‐off phase, and rose sharply to 1.667 after stimulation initiation; CC slightly increased from 0.727 pre‐operatively to 0.789 post‐operatively, decreased to 0.652 in the stimulation‐off phase, and rebounded to 0.75 after stimulation initiation; Dens increased from 0.733 pre‐operatively to 0.80 post‐operatively, fell back to 0.60 in the stimulation‐off phase, and continued to decline to a stable level of approximately 0.533 after stimulation initiation; Str decreased from 0.538 pre‐operatively to 0.478 post‐operatively, rose slightly to 0.485 in the stimulation‐off phase, and increased to approximately 0.525 after stimulation initiation. In contrast, the motor network displayed far more pronounced and drastic fluctuations across all phases, with several metrics (e.g., APL in the stimulation‐on phase) deviating significantly from the pre‐operative baseline. (b) Intra‐network connectivity strength and network Segregation Index: Analyses of intra‐network connectivity and segregation for the cognitive and motor networks revealed that the intra‐network connectivity strength of the cognitive network was approximately 0.502 pre‐operatively, remained nearly unchanged at 0.503 post‐operatively, rose to 0.60 in the stimulation‐off phase, and further decreased to 0.508 (still higher than the pre‐operative level) after stimulation initiation, presenting an overall fluctuation trend of stable post‐operatively, elevated in the stimulation‐off phase, and slightly decreased yet still higher than the pre‐operative level after stimulation initiation. For the motor network: The intra‐network connectivity strength was approximately 0.538 pre‐operatively, dropped sharply to 0.479 post‐operatively, rose slightly to 0.483 in the stimulation‐off phase, and continued to increase to approximately 0.522 after stimulation initiation. Unlike the cognitive network, its connectivity strength exhibited a fluctuation pattern of an initial decrease, subsequent increase, and further elevation after stimulation initiation. Additionally, the dynamic changes in the Segregation Index further confirmed the relative relationship between the two networks [Segregation Index = (cognitive network connectivity strength − motor network connectivity strength) / (cognitive network connectivity strength + motor network connectivity strength)], which reflects the degree of relative independence between the two networks (a value closer to 1 indicates a higher level of mutual independence between the networks). The Segregation Index was approximately 1.05 pre‐operatively (indicating a relatively independent cognitive network), decreased to −0.05 post‐operatively (the motor network became dominant), rebounded to 1.04 in the stimulation‐off phase, and slightly declined to 1.01 after stimulation initiation (the cognitive network regained dominance). The connectivity strength of the two networks exhibited significant inverse fluctuations (the cognitive network decreased when the motor network increased, and vice versa), and the Segregation Index was dynamically adjusted across different phases. This asynchronous and divergent change pattern further confirmed that the functional reorganization of the cognitive network is an active process independent of the motor network, rather than a passive outcome mediated by the remodeling of the motor network, effectively excluding the confounding effects of the motor network on the core research focus of the cognitive network. (1) GE (Global Efficiency): Measures the overall efficiency of information transmission in the network; higher values indicate faster information propagation; (2) APL (Average Path Length): Represents the average “number of steps” for information transmission between nodes; lower values imply better network integration; (3) CC (Clustering Coefficient): Quantifies the degree of aggregation of local connections around nodes; higher values denote tighter local connectivity within the network; (4) Dens (Density): Refers to the ratio of actual connections to the maximum possible connections in the network; higher values indicate a denser network structure; (5) Str (Strength): Represents the sum of weights of all connections of a node; higher values signify stronger overall connectivity strength of the node.

In this study, cognitive function‐related regions of interest (ROIs) were defined as the core cognitive network, whose components and corresponding fNIRS channels are as follows: (1) Default Mode Network (DMN): Including the mPFC, covered by Ch 7, 8, 9, 44; (2) Dorsal Attention Network (DAN): Including the DLPFC, covered by Ch 4, 5, 6, 10, 11, 12, 23, 24, 25, 26, 27, 28, 29, 30, 41, 47; (3) Ventral Attention Network (VAN): Including the VLPFC (Ch 19, 20, 21, 22, 31, 32, 33, 34, 42, 49), TL (Ch 18, 37, 38, 39, 40, 50, 51, 52), and IPL (Ch 1, 14, 16, 35). In addition, the core sensorimotor network was defined as the bilateral SMA, covered by Ch 2, 3, 13, 15, 17, 36. After defining the fNIRS channels corresponding to the above ROIs, graph theory approaches were applied to calculate changes in graph‐theoretic metrics (including intra‐network connectivity strength, global efficiency, and local efficiency) for the core cognitive network and the sensorimotor network, respectively, during the perioperative period of DBS (T0, T1, T2, T3). Subsequently, the amplitude and pattern of changes within these two networks were compared to verify their independence, and the Segregation Index was calculated to characterize alterations in cognitive network connectivity and explore the potential induced reorganization of the cognitive network after DBS surgery and electrical stimulation initiation. The sensorimotor network was isolated to exclude its potential confounding effects on the cognitive network, thereby investigating the DBS‐induced reorganization of the cognitive network independently. For the graph theory model: each node corresponds to a single fNIRS channel (each channel represents a specific brain region); each edge corresponds to the functional connectivity strength between two brain regions (typically quantified by the Pearson correlation coefficient, where a higher correlation coefficient indicates a greater edge weight). To further quantify the dynamic reorganization of cognitive and motor core networks during the perioperative period of bilateral STN‐DBS, graph theory approaches were applied to analyze changes in topological properties of two core networks—the cognitive network (including DMN, DAN, VAN, TL, and IPL) and the SMA—derived from task‐state fNIRS data. Nodes in the networks were mapped to fNIRS channels, and edge weights were calculated based on the strength of functional connectivity between hemodynamic signals of different channels. (1) Changes in network topological properties: The topological properties of the cognitive network exhibited a mild yet sustained optimizing trend during the perioperative period (Figure 5a). Compared with the Pre‐operative stage (T0), both the global efficiency (GE) and clustering coefficient (CC) of the network slightly increased at the Post stage (T1), indicating a transient enhancement in local information processing capacity. These metrics essentially returned to Pre levels during the Off stage (T2). After stimulation initiation (On, T3), the global efficiency of the cognitive network was further elevated to 0.81, while the average shortest path length (APL) decreased to 1.48, demonstrating a significant optimization in the overall information transmission efficiency and an enhancement in the integrative capacity of the network. In contrast, the motor network displayed more pronounced stage‐dependent fluctuations. Its GE and Dens transiently increased at the post‐operative microlesion stage (T1), which may be associated with altered local activation patterns induced by acute surgical effects. However, after stimulation initiation (T3), the APL increased significantly to 1.40, whereas the CC and Dens decreased relative to the pre‐operative baseline, suggesting that the motor network may undergo a more decentralized or specialized reorganization pattern under long‐term electrical stimulation. (2) Changes in intra‐network connectivity strength further revealed the segregation and reorganization characteristics of the two systems (Figure 5b): The connectivity strength of the cognitive network remained stable at the post‐operative microlesion stage (T1), and although it slightly decreased after stimulation initiation, it still remained higher than the pre‐operative level, presenting a trend of postoperative stability followed by slight modulation after stimulation. In contrast, the connectivity strength of the motor network decreased markedly post‐operatively and then gradually recovered after stimulation initiation, showing a dynamic trajectory of initial suppression followed by recovery. Furthermore, to quantify the independence of the two networks, we calculated the Segregation Index. This index was 1.05 pre‐operatively, indicating a relatively independent cognitive network; it decreased to 0.97 at the post‐operative microlesion stage, suggesting a transient dominance of the motor network; and it recovered to 1.01 after stimulation initiation, with the cognitive network regaining its dominant status. This inverse fluctuating pattern of reciprocal changes indicated that the functional reorganization of the cognitive network during the STN‐DBS perioperative period is an active process independent of the motor network, rather than a passive reflection of the reorganization of the latter. In summary, graph theory‐based fNIRS analysis revealed that the cognitive network gradually evolves toward higher efficiency and enhanced integration after STN‐DBS, whereas the motor network undergoes more drastic stage‐dependent reorganization. The two networks exhibited complementary fluctuations in connectivity strength and Segregation Index, which further confirmed the independence and specificity of the functional reorganization of the cognitive network. These findings provide quantitative evidence for understanding the modulatory mechanisms of STN‐DBS on non‐motor networks. Detailed changes in all indices are presented in Figure 5.

## 4 Discussion

This study employed fNIRS to longitudinally track the dynamic changes in cerebral cortical activation in patients with PD during the performance of the VFT across the perioperative period of bilateral STN‐DBS (Pre, Post, Off, On). We revealed that the prefrontal‐temporal cognitive‐related networks underwent an evolutionary process from acute postoperative inhibition to late‐stage functional reorganization. Notably, graph theory analysis demonstrated that the reorganization of this cognitive network exhibited characteristics independent of the motor network, and electrical stimulation may have played a positive role in network modulation and optimization during this process. These findings provide novel, high‐temporal‐resolution evidence for understanding the neural mechanisms of the MLE and the neurocognitive modulatory effects of STN‐DBS.

### 4.1 Microlesion Effect: Acute Local Disruption of Cognitive Networks

One of the most striking findings of this study is the widespread suppression of cortical activation during the postoperative MLE phase (T1). Compared with the preoperative period (T0), the number of significantly task‐related activated channels sharply decreased from 8 to 2, accompanied by a significant decline in patients' VFT performance. This provides intuitive hemodynamic evidence for the transient cognitive dysfunction observed clinically. From a functional perspective, the loss of activation was primarily concentrated in key nodes supporting language and executive functions [41]: activations in the VLPFC (particularly Broca's area) and DLPFC nearly completely disappeared. These regions are core to lexical retrieval, language production, and cognitive control [42]. Furthermore, intergroup comparisons between T1 and T0 revealed a significant reduction in activation of the right temporal lobe (TL) (Ch40) during T1, indicating that the surgical impact extended beyond the prefrontal cortex to involve posterior components of the fronto‐temporal language network. These findings are consistent with previous literature, further confirming the critical role of the fronto‐temporal network in verbal fluency and executive function [43, 44]. Notably, during the natural recovery phase (T2, 1 month postoperatively without electrical stimulation), patients' behavioral performance and activation of certain brain regions had significantly improved compared with the postoperative MLE phase (T1). Furthermore, these findings may support the hypothesis that MLE is not only a direct effect of electrode implantation on local STN [45], but more likely induces transient “functional disconnection” in the traversing cortico‐basal ganglia‐thalamo‐cortical loops. Our results suggest that surgical‐related MLE may lead to functional inactivation of large‐scale fronto‐temporal cognitive networks supporting complex executive tasks [46, 47], thereby explaining the clinically observed transient cognitive dysfunction. Importantly, this cognitive network inactivation cannot be fully reversed over time, indicating a chronic, mild, and persistent impact of surgical intervention on cognitive circuitry.

### 4.2 Potential Role of Bilateral STN‐DBS in Cognitive Network Optimization

Notably, after the initiation of DBS stimulation (T3), we observed a more extensive cortical activation pattern compared with the pre‐operative baseline (T0). The number of activated channels recovered and exceeded the pre‐operative level, reaching 12, covering a wide range of regions including the VLPFC, DLPFC, SMA, and temporal association cortex. Intergroup comparison results (T3 vs. T2) further confirmed these findings: following electrical stimulation, the activation levels of multiple key cognitive brain regions—including the bilateral VLPFC and left DLPFC—were significantly higher than those in the natural recovery phase (T2). This indicates that the intervention of electrical stimulation may “additionally” enhance or reshape the functional involvement of these core brain regions beyond the plateau achieved by natural recovery. This “rebound” phenomenon occurred synchronously with the normalization of VFT behavioral performance, suggesting that DBS stimulation (On) may promote functional reorganization within the cognitive network [48]. Meanwhile, this “additional” modulatory effect was echoed by graph‐theoretic metrics. Compared with T2, the GE of the cognitive network in T3 was further elevated (0.811 vs. 0.752), and the APL was further shortened (1.506 vs. 1.379), indicating that the overall information transmission efficiency and integration capacity of the network were further optimized under electrical stimulation. Additionally, the significant increase in network Dens (0.509 vs. 0.621) suggested the establishment of richer functional connections within the network. The synchronous improvement of these topological metrics collectively depicts a picture of the cognitive network evolving into a more efficient, integrated, and tightly connected “small‐world” network after electrical stimulation. Previous relevant studies have reported that the recovery of cognitive function during the postoperative MLE phase is associated with the resolution of surgical trauma rather than the initiation of stimulation [49]. However, the dynamic changes in the fronto‐temporal cortical cognitive network induced by electrode implantation (MLE) and subsequent stimulation observed in this study cannot be attributed solely to the recovery of surgical trauma. Instead, bilateral STN‐DBS may exert an interfering and reorganizing effect on the cognitive network of PD patients. Previous studies have shown that DBS has a clear effect on regulating motor network circuits to improve motor symptoms, and it also has potential regulatory effects on cognitive networks that can be observed through clinical symptoms [50, 51]. Therefore, the regulatory effect of bilateral STN‐DBS on cognition is confirmed but not yet fully understood, and its potential pathway basis may be precisely the STN‐prefrontal connections depicted in healthy populations [17]. MLE may temporarily interfere with the normal information transmission of these circuits, leading to the inactivation of cortical cognitive nodes; in contrast, DBS stimulation may modulate the output of the STN, thereby exerting neuromodulation on the activity of the upstream prefrontal cortex along these existing anatomical connections, ultimately promoting the functional reorganization of the cognitive network. Our study supports this potential mechanism.

### 4.3 Specific Reorganization of the Cognitive Network During the Perioperative Period of DBS: Evidence for Independence From the Motor Network

A previous study demonstrated that motor network oscillatory activity after bilateral STN‐DBS is primarily associated with *β* oscillations, whereas cognitive network oscillatory activity is not confined to a single frequency band but involves multiple frequency bands collectively [52]. This raises a critical question: Are the STN‐DBS‐induced changes in the cognitive cortical network cognitively independent and specific, or are they systemic effects secondary to improved motor symptoms, or merely accompanying phenomena of motor network remodeling? The present study provides preliminary evidence through systematic intergroup comparisons and graph theory analysis: (1) First, regarding intergroup differences in activation patterns, comparisons between the stimulation‐on group (T3) and the postoperative MLE group (T1) revealed significantly enhanced activation in regions including the right DLPFC (Ch6), right VLPFC (Ch19), and left VLPFC (Ch47). This specific enhancement in activation patterns points to cognitive networks closely related to executive control and language production. More importantly, direct comparisons between the stimulation‐on group and the pre‐operative baseline (T3 vs. T0) showed that the activation intensity of the left DLPFC (Ch30) after stimulation even exceeded the pre‐operative level. This strongly suggests that electrical stimulation may not merely “restore” network function to the baseline, but may induce functional enhancement or configurational optimization beyond the baseline; (2) Second, graph theory analysis provided quantitative evidence at the network level. Results showed that the evolutionary trajectories of topological properties of the cognitive and motor networks during the perioperative period were asynchronous: the global efficiency of the cognitive network continued to optimize after stimulation initiation, while the motor network exhibited greater volatility and distinct remodeling patterns during the same period. The most compelling evidence comes from the dynamic changes in the Segregation Index—this index shifted from cognitive dominance pre‐operatively (1.05) to transient motor network dominance during the postoperative MLE phase (0.979) and ultimately to clear cognitive network re‐dominance after stimulation initiation (1.012). This U‐shaped trajectory of “cognitive dominance → motor dominance → cognitive re‐dominance” was highly consistent with the trend of cognitive behavioral scores (VFT): “decline → partial recovery → further improvement”. These findings collectively demonstrate that STN‐DBS‐induced cortical functional reorganization is not a homogeneous global event; instead, the cognitive network undergoes a remodeling process with relative independence and specificity. This provides empirical support from task‐state network dynamics for the theory that “the STN is a key node integrating motor and non‐motor information”, and also suggests that the therapeutic mechanism of DBS may exert differential modulation on motor and non‐motor symptoms through parallel but partially segregated circuits—consistent with the results of a previous study [53].

### 4.4 Stage‐Specific Mechanisms Revealed by Behavioral‐Neural Correlations

In the present study, correlation patterns between behavioral performance and brain region activation were observed to exhibit a clear stage‐specific evolution. After FDR correction, these specific correlations provide key insights into the functional consequences of network reorganization. This shift from a positive correlation pattern to a complex negative correlation pattern may reflect the dynamic process of neural networks evolving from a state of “effortful compensation” to “efficient operation” under external intervention [54]. (1) At the pre‐operative baseline (T0), a significant positive correlation was observed between VFT performance and the activation of the left DLPFC, bilateral TL, and left VLPFC. This is consistent with classic cognitive neuroscience models, wherein superior executive function and verbal fluency rely on more robust coordinated activation of the prefrontal control network and temporal semantic network [55, 56]. However, the simultaneously observed negative correlation between MoCA scores and the activation of the right DLPFC and mPFC may reflect a characteristic of early cognitive impairment in PD: prefrontal cortical dysfunction may have already occurred, and additional “overactivation” is required to maintain cognitive levels at this stage. Nevertheless, this overactivation itself may be inefficient or compensatory and inconsistent with optimal overall cognitive status, suggesting that the cognitive network of PD patients is not in a state of optimal efficiency even pre‐operatively [57]; (2) During the postoperative microlesion phase (T1), a fundamental reorganization of correlation patterns was observed. With the widespread inhibition of core cognitive nodes such as the DLPFC and VLPFC, VFT performance was instead positively correlated with the activation of the left sensorimotor area and right superior temporal gyrus. This finding further supports the “neural compensation” hypothesis [58], wherein when the higher‐order cognitive control network (prefrontal cortex) is functionally impaired due to acute surgical effects, the brain strategically relies more on relatively preserved, more basic sensorimotor integration loops and semantic storage/comprehension networks (posterior temporal lobe) to complete the task [59]; (3) During the postoperative natural recovery phase (T2), the correlation pattern evolved further. VFT performance remained positively correlated with the bilateral IPL, and the left SMA, indicating that these pathways remained important components of the cognitive executive network during the natural recovery phase [60]. However, the correlation between MoCA scores and left temporal lobe activation shifted to a negative one. This negative correlation may imply that, at the plateau achieved by natural recovery, the persistent, relatively rigid or inefficient overactivation of the left temporal lobe may limit the further development of the overall cognitive network toward a more optimized configuration [61]; (4) The most theoretically significant shift occurred after stimulation initiation (T3). At this stage, a significant negative correlation was observed between VFT performance and the activation of the right DLPFC and left sensorimotor area. This phenomenon cannot be simply interpreted as a reduction in the function of these brain regions. Instead, when combined with the superior behavioral performance, more specialized and extensive activation pattern, and enhanced global network efficiency revealed by graph theory analysis in T3, these findings collectively point to the “neural efficiency optimization hypothesis [62]”. Following chronic DBS modulation, the cognitive network (particularly the prefrontal executive control system) may have completed functional reorganization, achieving more precise and economical information processing and resource allocation [63]. An efficient network can achieve the same or even better behavioral output with lower overall activation cost or a more specific activation pattern through closer coordination between nodes and more optimized information routing [64]. The negative correlation between right DLPFC activation and behavioral performance may reflect its role as a cognitive control hub—its interaction efficiency with other network nodes (e.g., VLPFC, temporal lobe) has been significantly improved, thereby effectively regulating task progression without the need for high‐intensity local activation [65]. In summary, this dynamic trajectory of behavioral‐neural correlation patterns—from relying on core cognitive brain regions (positive correlations), to shifting dependence on sensorimotor and posterior semantic regions for compensation (positive correlation transfer), and ultimately evolving into efficient negative correlations with key prefrontal regions—clearly depicts a continuous pathway of neural networks progressing from “inefficient compensation after acute injury” to “efficient reorganization following chronic modulation.” This pathway corroborates with the longitudinal changes in cortical activation patterns and network topological properties, forming an internally consistent evidence chain regarding how STN‐DBS induces phased remodeling of cognitive neural circuits in PD patients. These findings provide new insights into the potential mechanisms underlying the cognitive benefits of DBS.

It should be noted that the present study has certain limitations [66]. First, fNIRS is inherently constrained in spatial resolution, with its effective detection depth typically limited to the cerebral cortex (approximately 2–3 cm), which precludes the direct recording of neural activity in subcortical deep nuclei including the STN, hippocampus, and basal ganglia [19]. Thus, the activation and reorganization patterns of the prefrontal‐temporal cortical network observed in this study are essentially downstream effects or distal network responses induced by STN‐DBS intervention, rather than real‐time monitoring of the STN or the deep neural circuits with which it forms direct connections. This technical limitation is critical for investigating the underlying mechanisms of DBS. Numerous studies employing deep brain recording or high‐spatial‐resolution imaging techniques (e.g., DTI, fMRI) have confirmed that the STN establishes direct and indirect structural and functional connectivity with extensive cortical regions (including the prefrontal cortex) and is closely implicated in cognitive processing [16, 67]. Similarly, investigations into the role of medial temporal lobe structures such as the hippocampus in memory and cognitive function have largely relied on fMRI, PET, or intracranial electrophysiological recordings [68]. While these techniques can provide critical insights into the neural activity of deep nuclei, they often involve inevitable trade‐offs in temporal resolution, portability, or invasiveness. In contrast, the core advantages of fNIRS lie in its high temporal resolution, non‐invasiveness, resistance to motion artifacts, and ease of bedside application—attributes that render it highly suitable for the dynamic monitoring of cortical functional plasticity during longitudinal, task‐based experimental paradigms. The value of the present study does not reside in the direct elucidation of the local electrophysiological properties of the STN, but rather in the high‐temporal‐precision characterization of the evolutionary trajectory of the cortical cognitive network over the STN‐DBS perioperative period, which provides a unique perspective for understanding the distal network effects of DBS. Second, the study lacked a healthy control group, which precludes the direct characterization of the features of the STN‐cortical connectivity network in PD patients relative to healthy individuals. Future studies should enroll a sufficient number of healthy controls and integrate resting‐state fMRI (rs‐fMRI) with DTI to construct brain network atlases, thereby further clarifying the baseline abnormalities of the cognitive network in PD patients and the specific modulatory effects of STN‐DBS on these aberrant networks. Furthermore, although the present study disentangled the effects of natural post‐surgical recovery from those of electrical stimulation by adding the T2 time point, it was still constrained by clinical ethical principles and standard operational protocols, which precluded the adoption of a delayed stimulation design for longer‐term follow‐up assessments. Future research should employ more precise and refined grouping strategies and recruit a larger cohort of patients to further validate the findings of the present study.

## 5 Conclusion

In summary, through longitudinal fNIRS dynamic monitoring, this study systematically delineated the evolutionary trajectory of the prefrontal‐temporal cognitive network in PD patients following bilateral STN‐DBS treatment, from acute inhibition to functional reorganization. Further graph theory‐based network analysis confirmed that the reorganization of the cognitive network exhibits characteristics independent of the motor network. The results indicate that the improvement in postoperative cognitive function is a multi‐stage process: natural recovery after acute surgical effects forms the basis for functional rebound, while STN‐DBS electrical stimulation may further exert a positive neuromodulatory effect on this foundation, promoting the optimization of the cognitive network toward a more efficient and integrated state. This study verifies the unique value of fNIRS as a non‐invasive, portable, and practical bedside tool for monitoring DBS‐induced cortical plasticity, providing novel, high‐temporal‐resolution evidence for an in‐depth understanding of the complex effects of DBS on cognitive function. Future research should focus on multi‐modal technology integration, more refined temporal design, and long‐term follow‐up to fully clarify the underlying neural mechanisms and guide personalized treatment.

## Author Contributions

**Xiang Wei:** software, writing – original draft, conceptualization, investigation, data curation, and methodology. **Yuting Tian:** writing – original draft, methodology, data curation, investigation, and validation. **Qiutian Lu:** data curation, investigation, and methodology. **Jingxuan Liu:** data curation and investigation. **Guanghan Lu:** data curation, investigation, and methodology. **Jian Sun:** data curation and methodology. **Bei Luo:** data curation and methodology. **Liang Zhao:** writing – review and editing and formal analysis. **Chang Qiu:** writing – review and editing, formal analysis, and resources. **Wenbin Zhang:** conceptualization, writing – review and editing, funding acquisition, validation, and supervision. **Wenwen Dong:** writing – review and editing, conceptualization, funding acquisition, validation, and supervision.

## Funding

This work was supported by the Jiangsu Provincial Key Research and Development Program (Grant Nos. BE2022049 and BE2022049‐1); the Promotion and Application Project of High‐Field MRI‐Compatible Deep Brain Stimulators (2024 High‐End Medical Equipment Promotion and Application Project), funded by the Ministry of Industry and Information Technology (MIIT) and the National Health Commission (NHC) of the People's Republic of China (Grant No. 2024TGYY46); and the Nanjing Medical University Science and Technology Development Fund Project (NMUB20250184).

## Ethics Statement

This research was conducted in accordance with the Declaration of Helsinki and received ethical approval from the Ethics Committee of Nanjing Medical University Affiliated Brain Hospital (Approval No: 2022‐KY005‐01). All patients provided written informed consent.

## Conflicts of Interest

The authors declare no conflicts of interest.

## Data Availability

The relevant data used in this study are available from the corresponding authors upon request.
